# Tiny Toes to Tau Tangles: Down's Syndrome and Its Association With Alzheimer's Disease

**DOI:** 10.7759/cureus.22125

**Published:** 2022-02-11

**Authors:** Sri Madhurima Puttagunta, Rabia Islam, Sumana Kundu, Surajkumar B Jha, Ana P Rivera, Gabriela Vanessa Flores Monar, Hamza Islam, Ibrahim Sange

**Affiliations:** 1 Research, Dr. Pinnamaneni Siddhartha Institute of Medical Sciences, Chinoutpalli, IND; 2 Research, Faisalabad Medical University, Faisalabad, PAK; 3 Research, R. G. Kar Medical College, Kolkata, IND; 4 Research, Jinan University School of Medicine, Guangzhou, CHN; 5 Research, Universidad Americana (UAM) Facultad de Medicina, Managua, NIC; 6 Research, Universidad Central del Ecuador, Quito, ECU; 7 Research, K. J. Somaiya Medical College and Research Centre, Mumbai, IND

**Keywords:** alzheimer's disease, chromosome 21, alzheimer’s dementia, amyloid beta protein, acetylcholinesterase inhibitors, amyloid plaques, trisomy 21, intellectual disability (id), amyloid precursor protein, down's syndrome

## Abstract

Down’s syndrome (DS) is a common genetic condition caused by the trisomy of chromosome 21, which leads to the development of many multisystemic complications, early-onset Alzheimer’s disease (AD) being one of its most common complications. In this article, we have performed an intensive literature review that established a strong relationship between AD and DS. These two conditions are clubbed pathologically, clinically, and diagnostically to understand the association between AD and DS. This article focuses on understanding the impact of AD on a DS patient on both clinical and pathological levels and exploring some advanced treatment modalities. It has also emphasized the importance of early screening and diagnosis for AD in this group of patients to prevent AD development. Regular monitoring, early diagnosis, and a proper treatment plan can slow the AD occurrence in DS patients.

## Introduction and background

Down's syndrome (DS) is the most common human genetic alteration and a common cause of intellectual disability (ID), which is caused due to aneuploidy of chromosome 21 [1,2]. The English physician John Langdon Down first described DS in 1862 [3]. In 1959, Jerome Lejeune identified that DS was caused by the presence of extra chromosomes [3,4]. According to 2010 statistics, DS occurs in one per 1000 births, and according to 2015 statistics, 5.4 million individuals suffered from DS globally, leading to 27,000 deaths [5,6]. DS equally affects both males and females of all races, and clinical presentation is variable with the ethnicity of the patient [7]. DS can develop due to factors associated with the paternal origin or maternal origin, but factors related to maternal origin are the main contributing factor [8]. The risk factors of DS mainly focus on maternal causes, and it is primarily related to advanced maternal age at conception [8].

DS mainly occurs through three pathologic defects: (a) full trisomy of chromosome 21, (b) mosaicism of chromosome 21, and (c) inheritance of a structural rearrangement that causes partial trisomy of the majority of content [8]. Other causes like Robertsonian translocation, isochromosome, and ring chromosome can also cause DS [3]. DS causes many clinical symptoms that result from cognitive, physical, and physiological impairment [2]. It can result in complications like congenital heart diseases, epilepsy, leukemia, thyroid diseases, ID, AD, Hirschsprung disease, etc. [3,9].

Ultrasound (US) in 14-24 weeks of gestation and blood tests in the second trimester can be used as screening tests to detect DS prenatally, and findings from the above-mentioned screening tests can be confirmed by amniocentesis and chorionic villus sampling [10-12]. The management approach for patients with DS mainly focuses on early intervention by methods like speech therapy, physiotherapy, occupational therapy, and proper medical attention for different health issues [13]. The most prevalent type of dementia is AD, and the leading genetic risk factor for early-onset AD is DS [14].

According to an estimate, there are 250,000 to 400,000 DS patients in the United States, and nearly all can start to develop AD in their 30s [15]. In DS patients, cognitive assessment is challenging, and standard domestic methods cannot be used, making early diagnosis difficult [16]. The review aims to understand the relationship between DS and AD and the effect of AD in adult DS patients and to explore all the management modalities available for adult DS patients who have associated AD.

## Review

The largest groups of people with dementia below 50 years are individuals with DS, which is the common genetic cause of learning difficulties [16]. To explore the state of research on AD and DS, Alzheimer’s Association, the global DS foundation, and LuMind IDSC Foundation, Burlington, Massachusetts, partnered and conducted a workshop in March 2019. In this workshop, they discussed the unmet needs and research gaps in the area of AD and DS patients and the best ways to advance in this topic and also to identify deficits in our understanding for future focus and many more [17].

The pathological bridge between AD in DS

The upstream molecule in AD pathogenesis is beta-amyloid (A-B) protein [18]. DS patients present with AD-like pathogenesis in the early 40s compared to sporadic patients [19]. This is because of a 1.5-fold higher amyloid precursor protein (APP) expression in DS patients, which results in a 1.5-fold increase in A-B production [19]. Mapping the APP gene, which encodes for A-B, takes us to chromosome 21, and DS is caused by the third copy of chromosome 21 [20,21]. Thus this increase in gene dosage is the biggest neuropathogenic culprit in AD and DS patients [20].

**Figure 1:**
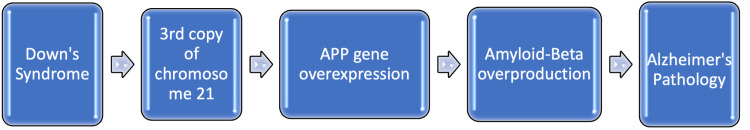
Basic scheme of development of AD in DS patients APP: Amyloid precursor protein; AD: Alzheimer’s disease; DS: Down’s syndrome.

The neuropathologic phenotype of older DS patients closely resembles AD patients [22]. In 1996, Lemere et al. conducted a study to examine DS subject brains and provide a model for the pathogenesis of AD [22]. Twenty-nine DS subjects between the age of three and 73 years were considered [22]. In this study, with the help of computerized image analysis, the amyloid plaque number and the percentage of the cortical area they occupied were quantified [22]. They identified that the A-B 42 amino acid was deposited at a young age, around 7-16 years, and A-B 40 amino acid was started to detect around the age of 30 [22]. They also found that A-B 42 immune reaction plaques were abundant compared to A-B 40 immune reaction plaques, even in the individuals that were 51-73 years old [22]. This study concluded that the formation of A-B 42 ending peptides begins at a young age in DS patients, but the number and percentage of the cortical area of A-B 42 plaques increase only a little with advanced age (Table 1) [22]. The summary of the development of AD in a DS patient is illustrated in Figure 1.

**Table 1:** Summary of studies showing a pathological correlation between AD and DS AD: Alzheimer’s disease; DS: Down’s syndrome; A-B: amyloid-beta protein.

Reference	Year	Design	Population	Variable	Conclusion
Flores-Aguilar et al. [26]	2020	Cross-sectional study	127 subjects, 16 gestation weeks – 64 years	Down’s syndrome	Microglial and inflammatory changes observed from an early age in DS patients
Coppus et al. [25]	2009	Prospective study	394 subjects	Down’s syndrome	High levels of neopterin concentration seen in demented DS patients
Lemere et al. [22]	1996		29 subjects, 3-73 years	Down’s syndrome	A-B 42 ending peptide formation begins at an early age in DS patients

The early initiating factor of AD is the imbalance between the production and clearance of A-B 42 and related A-B peptides [23]. The catalytic site of gamma-secretase is presenilin [23]. Therefore, all dominant mutations, either in the substrate (APP) or the protease (presenilin), can cause early-onset AD [23]. A major A-B degrading enzyme, neprilysin, is also degraded in DS patient-derived fibroblasts; this decreasingly ineffective regulation of neprilysin occurs due to dual-specificity tyrosine phosphorylation-regulated kinase 1A (DYR1A) overexpression [19,24]. Neopterin, a marker for cell-mediated immune activation and inflammation, can be identified as a risk factor for dementia in DS patients [25]. A prospective study was conducted by Coppus et al. in 2009 to evaluate neopterin level and the association in DS patients [25]. They considered 394 subjects with DS for their study, and the risk of dementia was determined by the Cox proportional hazard model [25]. They observed high plasma neopterin levels in demented subjects with DS compared to non-demented subjects with DS [25]. They concluded that higher neopterin levels in plasma were also a risk determinant of dementia in DS patients (Table 1) [25].

The possibility of cytokinin involvement in the development of early events of AD pathogenesis is supported by identifying neuroinflammatory changes such as the proliferation of activated glia, immune cytokinin Interleukin-1 (IL-1), and S-100 in brains of fetuses, neonates, and children [20]. Flores-Aguilar et al. conducted a cross-sectional study in 2020 to study the evolution of neuroinflammation across AD in DS patients [26]. A total of 127 DS subjects ranging from 16 weeks of gestation to 64 years were considered in this study [26]. They used techniques like immunohistochemistry and electrochemiluminescent-based immunoassay in the frontal cortex of study subjects to identify microglial morphology and inflammatory cytokinin expression [26]. Microglial morphologic changes such as an increase in microglial soma size to process length ratio and an increase of rod-like microglia are observed in the frontal cortex of children and young adults [26].

Inflammatory changes like an increase in the levels of interleukin-8 and interleukin-10 are seen in one- to 10-year-old children with DS [26]. Increase in the levels of interleukin-1 beta, interleukin-1 alpha, interleukin-6, interleukin-8, interleukin-10, interleukin-15, eotaxin-3, and interferon-gamma-induced protein 10 are seen in subjects between 13 and 25 years [26]. Subjects from 59 to 62 years showed decreased levels of interleukin-10, interleukin-12P40, interferon-gamma, and tissue necrotizing factor-alpha [26]. All the above findings concluded that there is a presence of early and evolving neuroinflammatory phenotypes across the lifespan of DS patients (Table 1) [26]. Studies that support AD development in DS patients are summarized in Table 1.

The clinical liaison between AD and DS

National Institute of Aging and Alzheimer’s Association (NIA-AA) Research Framework defined AD by underlying pathology as measured in patients by biomarkers. For the staging of disease, clinical symptoms are used [27].

By the age of 40, all individuals with DS will develop the neuropathology of AD, and almost 60% will develop AD dementia symptoms by the age of 65 [28]. The AD evaluation involves structured patient and caregiver history taking [29]. A rapid deterioration in cognitive, adaptive, and behavioral functioning is experienced by a small percentage of adolescents and young adults with DS [30]. These include intellectual decline, loss of daily living skills, and significant behavioral changes [30]. This rapid deterioration is unexplained, and there is also no standardized workup to evaluate these patients [30]. Behavioral and psychosocial symptoms of dementia (BPSD) are the core symptoms of dementia. They include agitation, depression, apathy, psychosis, repetitive questioning, aggression, sleep problems, wandering, and inappropriate behaviors [31]. In DS patients, recognition of BPSD will increase the understanding of these behavior abrasions, thus focusing on adaptive caregiving and allowing for therapeutic intervention [32]. BPDS can be identified before the clinical AD diagnosis and can serve as an early indicator to identify the individuals at risk [32].

In individuals with DS and AD, neuropsychiatric symptoms (NPS) are frequent and contribute to caregivers’ distress [33]. In 2021, Fonseca et al. conducted a study on 92 individuals with DS to characterize NPS and caregiver distress among adults with DS [33]. All individuals are above 30 years, and they were divided into three subgroups: (1) stable cognition, (2) prodromal dementia, and (3) AD [33]. This categorization was made using Cambridge Examination for Mental Disorders of Older People With DS and Other Intellectual Disabilities (CAMDEX-DS) [33]. Participants underwent a neuropsychological assessment using the Cambridge Cognitive Examination [33]. They found that symptom severity varied from one group to another [33] and identified that agitation, apathy, and night-time behavior disturbance were associated with CAMCEX-DS [33]. Because of these symptoms, the caregivers’ distress was also impacted (Table 2) [33].

**Table 2:** Summary of studies mentioned in DS and AD clinical correlation NPS: Neuropsychiatric symptoms; DS: Down’s syndrome; AD: Alzheimer’s disease.

Reference	Year	Design	Population	Variable	Conclusion
Fonseca et al. [33]	2021		92 patients	Down’s syndrome	NPS is common in DS patients with AD, and it also causes distress to caregivers.
Fortea et al. [37]	2020	Cross-sectional study	388 cases, 242 controls	Down’s syndrome	In individuals with DS, AD biomarkers follow a predictable course over time.

**Figure 2:**
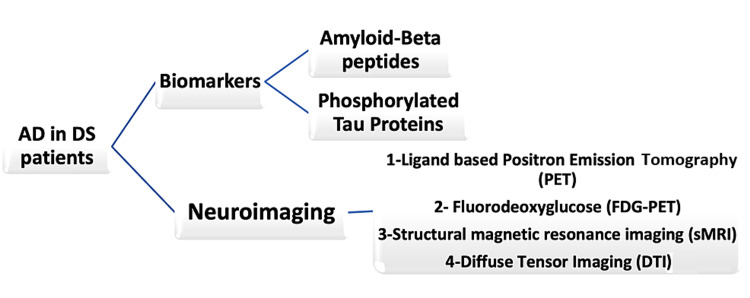
Diagnostic modalities for Alzheimer's disease in Down's syndrome patients AD: Alzheimer's disease; DS: Down's syndrome.

According to the recent work in both sporadic and autosomal dominant forms of AD, the amyloid pathology develops 15-20 years before neurodegeneration and symptomatic onset [34]. But tau pathology closely resembles symptomatic stages of cognitive decline and dementia [34]. The longitudinal assessment of spatial patterns in the accumulation of amyloid plaques and tau tangles in relation to symptomology can be identified by radiolabeled positron emission tomography (PET) [34]. Recently developed neuroimaging studies to analyze DS and AD are ligand-based PET, fluorodeoxyglucose PET (FDG-PET), and structural magnetic resonance imaging [35]. Emerging modalities are electroencephalography (EEG) and retinal imaging [35]. Neuron-derived exosomes are small extracellular vesicles secreted by cells in the body [36]. They contain A-B peptides and phosphorylated tau [36]. These can be used as blood biomarkers to predict dementia onset or progression in DS patients and are increased in DS patients with preclinical AD phase [36]. Different methods for diagnosis of AD in a DS patient are shown in Figure 2.

Fortea et al. conducted a dual center cross-sectional study in 2020 to identify the timing and order of changes in biomarkers of AD in DS patients [37]. For this study, 388 DS patients from Barcelona and Cambridge were recruited, and their cognitive impairment was classified with CAMCOG-DS [37]. Mild to moderate disability DS subjects were only considered, and they needed to have at least one of the AD measures [37]. These measures include apolipoprotein E allele carrier status, plasma concentrations of A-B peptides 1-42 and 1-40 and their ratio, total tau protein, and neurofilament light chain (NFL), tau phosphorylated at threonine 181 (p-tau) and NFL in cerebrospinal fluid (CSF), and one or more of PET with fluorodeoxyglucose, PET with amyloid tracers, and MRI [37]. A total of 242 controls for this study, who were cognitively healthy and euploid aged up to 75 years, were considered [37].

The earliest change was found in plasma NFL values around 30 years [37]. Around the fourth decade, they observed amyloid PET uptake [37]. Fluorodeoxyglucose PET and CSF p-tau changes were seen later in life, around the fifth decade [37]. It was concluded that AD has a long preclinical phase in DS individuals, and the biomarkers follow a predictable course of changes over time (Table 2) [37]. The summary of studies evaluating clinical and diagnostic modalities is depicted in Table 2.

Treatment modalities for AD in DS

Management mainly aims to delay and reduce cognitive decline, thus improving the quality of life [38]. But the management of AD in DS patients can be challenging due to underlying ID [39].

Pharmacological Treatment

For the identification of novel pharmacological therapies, a good understanding of the underlying pathophysiology of AD in DS is essential [40]. For symptomatic treatment of AD, cholinesterase inhibitors are widely used [41]. In 2002, Lott et al. conducted a non-randomized control trial using donepezil to find whether donepezil treatment would improve AD in DS patients [42]. They considered six treated patients and nine matched subjects as controls in their study and were administered donepezil for five months in the setting of Academic Medical Center [42]. In a period of three to five months, the dementia score in the targeted group was significantly improved [42]. This study concluded that the use of acetylcholine esterase inhibitors might help improve early and middle staged cognitive decline (Table 3) [42].

**Table 3:** Summary of studies included in the management modalities DS: Down’s syndrome; AD: Alzheimer’s disease; A-B: amyloid-beta.

Reference	Year	Design	Population	Conclusion
Potter et al. [44]	2021	Randomized control trial	20 subjects	Sargramostim showed a positive effect in the treated group compared to the control group.
Ptomey et al. [50]	2018		27 study participants	On cognitive function, exercise has a positive effect.
Cooper et al. [45]	2016	Randomized control trial		Changes in A-B level is less in patients treated with simvastatin.
Hanney et al. [46]	2012	Prospective, randomized double-blinded trial		Memantine is not adequate to treat AD in DS patients.
Boada et al. [47]	2012	Randomized, double-blind, placebo-controlled trial	40 study participants	Memantine shows no improvement in AD patients with DS.
Lott et al. [48]	2011	Randomized, double-blinded, placebo-controlled trial	53 participants	Antioxidant supplementation is not effective in treating dementia in DS patients.
Prasher et al. [43]	2002	Placebo-controlled trial	30 subjects were selected; 27 subjects completed data analysis	Donepezil has minor side effects in DS patients with AD.
Lott et al. [42]	2002	Non-randomized control trial	Six: treated patients; nine: controls	Acetylcholinesterase inhibitors show improvement in early and mild staged cognitive decline.

In another study conducted by Prasher et al. in 2002, they investigated the safety and efficacy of donepezil in DS adults [43]. It was a double-blinded, placebo-controlled study that was done for 24 weeks [43]. In the study, patients received a placebo or donepezil 5 mg per day during the first four weeks and 10 mg per day after that [43]. A total of 30 DS patients were selected, and 27 were included in data analysis [43]. In the donepezil group, 50% showed improvement with no harmful adverse effects, and in the placebo group, 31% showed improvement [43]. These results concluded that administering this drug once a day can be well-tolerated and safe in DS adults with AD (Table 3) [43].

Inflammatory markers seen in the cerebrospinal fluid and plasma of AD patients suggest contributing to AD and could be a therapeutic target [44]. In 2021, Potter et al. conducted a randomized, double-blinded, placebo-controlled trial to test if modulation of the innate immune system might be used to treat AD [44]. A total of 20 patients participated in this study [44]. They underwent treatment with sargramostim five days per week for three weeks, along with two follow-ups [44]. At the end of the treatment, the mini-mental state examination score increases in the sargramostim group compared to the placebo group [44]. After the treatment, the plasma markers of neurodegeneration also decreased in the sargramostim group compared to the placebo group (Table 3) [44].

Statins can delay AD onset by slowing amyloid deposition in the brain [45]. Cooper et al., in the year 2016, conducted a randomized control trial for 12 months using simvastatin 40 mg [45]. Fifty years and older population were considered in this study, but adults with dementia or simvastatin contraindication were excluded [45]. Blood markers A-B40 levels/A-B42 were measured along with cognitive function [45]. A total of 181 members were eligible, out of which 21 were recruited for the study [45]. Thirteen out of 21 patients completed the study in a year. A-B40 levels changed less for the simvastatin group (Table 3) [45].

Even though the prevalence of AD in DS people is strong, evidence to support the use of AD drugs in these people is very low [46]. Hanney et al. conducted a study in 2012 to see the safety and efficacy of memantine on cognitive function in DS patients [46]. This prospective randomized, double-blinded trial considered adults more than 40 years of age who are karyotype or clinically diagnosed with DS [46]. In this study, 88 participants received memantine, and 85 received a placebo [46]. Their outcome was measured by Down's syndrome attention, memory, executive function scales score (DAMES), and adaptive behavior scale (ABS) parts 1 and 2 [46]. Even though there was a decline in cognitive function, the rates did not differ between the groups for outcomes [46]. So, they concluded that memantine was not an effective treatment and that AD therapies are not necessarily effective (Table 3) [46]. Another randomized, double-blind, placebo-controlled trial was conducted by Boada et al. in 2012 to prove the hypothesis that memantine therapy would improve episodic and spatial memory in young adults with DS [47]. This study considered 40 young adults with DS, and all underwent treatment for 16 weeks either with memantine or placebo [47]. By the end of this study, they found no significant difference between the memantine and placebo groups, but a significant improvement in the memantine group was found in secondary measures (Table 3) [47].

Along with the risk of AD development in DS patients, they can also develop chronic oxidative stress [48]. Lott et al. conducted a randomized, double-blinded, placebo-controlled trial in 2011 to identify whether daily oral antioxidant supplementation was effective and safe in DS patients and dementia [48]. Fifty-three individuals were recruited for the study, and they received an antioxidant or placebo for two years [48]. They found that individuals who received antioxidant supplementation had neither improved cognitive function nor a stabilization of cognitive decline [48]. Through these findings, they concluded that antioxidant supplementation is safe but ineffective as a therapy for dementia in DS patients (Table 3) [48].

Non-pharmacological Treatment

Exercise intervention in an early stage of mild cognitive impairment (MCI), which is considered a preclinical stage in AD patients, can slow down the process of cognitive impairment in these patients, and it is also a cost-effective non-pharmacological therapy for dementia [49]. Ptomey et al. conducted a study in 2018 to evaluate changes in cognitive function in adults with DS [50]. This study was conducted with 27 participants, and they used to attend 30 minutes of group exercise sessions once or twice per week for 12 weeks [50]. Their cognitive function was measured at baseline and end of study [50]. Their study concluded that exercise has a positive impact on memory and other cognitive functions (Table 3) [50]. Studies supporting different pharmacological and non-pharmacological modalities for the treatment of AD in DS patients are depicted in Table 3.

Future Implications

There is very scarce evidence to prove the benefits of cholinesterase inhibitors and other therapeutic options to treat or delay the progression of the cognitive decline [16]. Despite close similarities with early-onset AD, individuals with DS respond differently to AD drugs [16]. So, a targeted approach for drug development is necessary [16]. Genetic and preclinical studies offer a great opportunity for treatment development, and by using this approach, potential therapies are being identified [16]. Even though amyloid cascade is involved in AD pathogenesis, removing toxic A-B alone is insufficient for disease modification [51]. A-B-centered clinical trials continue to dominate treatment strategies, and their value will be determined over time [51].

Limitations

DS and AD individually are very complex disorders, and a DS patient can develop many long-term risks, AD being one of them. In this article, we discussed AD pathogenesis, clinical features, and treatment concerned to a DS patient but not as an individual disease. Furthermore, this article did not cover all the long-term complications associated with DS and only focused on AD.

## Conclusions

The studies reviewed in this article clearly state that DS patients are prone to developing early-onset AD as early as in their 30s or 40s. In summary, the clinical implication of this article is to understand the pathological and clinical relationships of AD in DS patients and to use this knowledge to explore early diagnosis and treatment modalities to prevent the development of AD in DS patients. In this article, we specifically spoke about the importance of early diagnosis and the role of A-B and tau proteins as biomarkers in diagnosis. We also explored the benefits of acetylcholinesterase and statins as treatment modalities for the prevention of the development of AD. We believe that through this article, we can establish a unique connection between these two entities and understand the underlying difficulties for developing a better management approach. Even though recent studies mentioned in this article emphasized the success of acetylcholinesterase, statins, and failure of memantine as treatment options in these patients, a better and properly developed diagnostic and treatment plan is needed to identify the at-risk patients in their early stages and to prevent the development of disease. Lastly, we feel that in-depth research studies are required to be performed to construct a systemic management approach that helps to improve the quality of living in DS patients.

## References

[1] De Miguel A, De Miguel MD, Lucena-Anton D, Rubio MD (2018). [Effects of hypotherapy on the motor function of persons with Down's syndrome: a systematic review]. Rev Neurol.

[2] Arumugam A, Raja K, Venugopalan M, Chandrasekaran B, Sampath KK, Muthusamy H, Shanmugam N (2016). Down syndrome-a narrative review with a focus on anatomical features. Clin Anat.

[3] Hickey F, Hickey E, Summar KL (2012). Medical update for children with Down syndrome for the pediatrician and family practitioner. Adv Pediatr.

[4] Weijerman ME, de Winter JP (2010). Clinical practice. The care of children with Down syndrome. Eur J Pediatr.

[5] Lozano R, Naghavi M, Foreman K (2012). Global and regional mortality from 235 causes of death for 20 age groups in 1990 and 2010: a systematic analysis for the Global Burden of Disease Study 2010. Lancet.

[6] GBD 2015 Disease and Injury Incidence and Prevalence Collaborators (2016). Global, regional, and national incidence, prevalence, and years lived with disability for 310 diseases and injuries, 1990-2015: a systematic analysis for the Global Burden of Disease Study 2015. Lancet.

[7] Crawford D, Dearmun A (2016). Down's syndrome. Nurs Child Young People.

[8] Coppedè F (2016). Risk factors for Down syndrome. Arch Toxicol.

[9] Asim A, Kumar A, Muthuswamy S, Jain S, Agarwal S (2015). Down syndrome: an insight of the disease. J Biomed Sci.

[10] ACOG Committee on Practice Bulletins (2007). ACOG Practice Bulletin No. 77: screening for fetal chromosomal abnormalities. Obstet Gynecol.

[11] Agathokleous M, Chaveeva P, Poon LC, Kosinski P, Nicolaides KH (2013). Meta-analysis of second-trimester markers for trisomy 21. Ultrasound Obstet Gynecol.

[12] Alldred SK, Deeks JJ, Guo B, Neilson JP, Alfirevic Z (2012). Second trimester serum tests for Down's syndrome screening. Cochrane Database Syst Rev.

[13] Agarwal Gupta N, Kabra M (2014). Diagnosis and management of Down syndrome. Indian J Pediatr.

[14] Gomez W, Morales R, Maracaja-Coutinho V, Parra V, Nassif M (2020). Down syndrome and Alzheimer's disease: common molecular traits beyond the amyloid precursor protein. Aging (Albany NY).

[15] Hartley D, Blumenthal T, Carrillo M (2015). Down syndrome and Alzheimer's disease: common pathways, common goals. Alzheimers Dement.

[16] Ballard C, Mobley W, Hardy J, Williams G, Corbett A (2016). Dementia in Down's syndrome. Lancet Neurol.

[17] Snyder HM, Bain LJ, Brickman AM (2020). Further understanding the connection between Alzheimer's disease and Down syndrome. Alzheimers Dement.

[18] Zhang H, Zheng Y (2019). β amyloid hypothesis in alzheimer's disease: pathogenesis,prevention,and management. Zhongguo Yi Xue Ke Xue Yuan Xue Bao.

[19] Kawakubo T, Mori R, Shirotani K, Iwata N, Asai M (2017). Neprilysin is suppressed by dual-specificity tyrosine-phosphorylation regulated kinase 1A (DYRK1A) in Down-syndrome-derived fibroblasts. Biol Pharm Bull.

[20] Wilcock DM, Griffin WS (2013). Down's syndrome, neuroinflammation, and Alzheimer neuropathogenesis. J Neuroinflammation.

[21] Mazurek D, Wyka J (2015). Down syndrome--genetic and nutritional aspects of accompanying disorders. Rocz Panstw Zakl Hig.

[22] Lemere CA, Blusztajn JK, Yamaguchi H, Wisniewski T, Saido TC, Selkoe DJ (1996). Sequence of deposition of heterogeneous amyloid beta-peptides and APO E in Down syndrome: implications for initial events in amyloid plaque formation. Neurobiol Dis.

[23] Leuzy A, Chiotis K, Lemoine L, Gillberg PG, Almkvist O, Rodriguez-Vieitez E, Nordberg A (2019). Tau PET imaging in neurodegenerative tauopathies-still a challenge. Mol Psychiatry.

[24] Asai M, Kawakubo T, Mori R, Iwata N (2017). Elucidating pathogenic mechanisms of early-onset Alzheimer's disease in Down syndrome patients. Yakugaku Zasshi.

[25] Coppus AM, Fekkes D, Verhoeven WM, Evenhuis HM, van Duijn CM (2009). Neopterin and the risk of dementia in persons with Down syndrome. Neurosci Lett.

[26] Flores-Aguilar L, Iulita MF, Kovecses O (2020). Evolution of neuroinflammation across the lifespan of individuals with Down syndrome. Brain.

[27] Cohen AD, Landau SM, Snitz BE, Klunk WE, Blennow K, Zetterberg H (2019). Fluid and PET biomarkers for amyloid pathology in Alzheimer's disease. Mol Cell Neurosci.

[28] Rafii MS (2016). Improving memory and cognition in individuals with Down syndrome. CNS Drugs.

[29] Atri A (2019). The Alzheimer's disease clinical spectrum: diagnosis and management. Med Clin North Am.

[30] Jacobs J, Schwartz A, McDougle CJ, Skotko BG (2016). Rapid clinical deterioration in an individual with Down syndrome. Am J Med Genet A.

[31] Kales HC, Gitlin LN, Lyketsos CG (2015). Assessment and management of behavioral and psychological symptoms of dementia. BMJ.

[32] Dekker AD, Strydom A, Coppus AM (2015). Behavioural and psychological symptoms of dementia in Down syndrome: early indicators of clinical Alzheimer's disease?. Cortex.

[33] Fonseca LM, Mattar GP, Haddad GG (2021). Neuropsychiatric symptoms of Alzheimer's disease in Down syndrome and Its impact on caregiver distress. J Alzheimers Dis.

[34] Rafii MS (2019). Tau PET imaging for staging of Alzheimer's disease in Down syndrome. Dev Neurobiol.

[35] Neale N, Padilla C, Fonseca LM, Holland T, Zaman S (2018). Neuroimaging and other modalities to assess Alzheimer's disease in Down syndrome. Neuroimage Clin.

[36] Hamlett ED, Ledreux A, Potter H (2018). Exosomal biomarkers in Down syndrome and Alzheimer's disease. Free Radic Biol Med.

[37] Fortea J, Vilaplana E, Carmona-Iragui M (2020). Clinical and biomarker changes of Alzheimer's disease in adults with Down syndrome: a cross-sectional study. Lancet.

[38] Fonseca LM, Navatta AC, Bottino CM, Miotto EC (2015). Cognitive rehabilitation of dementia in adults with Down syndrome: a review of non-pharmacological interventions. Dement Geriatr Cogn Dis Extra.

[39] Prasher VP, Mahmood H, Mitra M (2016). Challenges faced in managing dementia in Alzheimer's disease in patients with Down syndrome. Degener Neurol Neuromuscul Dis.

[40] Caraci F, Iulita MF, Pentz R (2017). Searching for new pharmacological targets for the treatment of Alzheimer's disease in Down syndrome. Eur J Pharmacol.

[41] Larner AJ (2010). Cholinesterase inhibitors: beyond Alzheimer’s disease. Expert Rev Neurother.

[42] Lott IT, Osann K, Doran E, Nelson L (2002). Down syndrome and Alzheimer disease: response to donepezil. Arch Neurol.

[43] Prasher VP, Huxley A, Haque MS (2002). A 24-week, double-blind, placebo-controlled trial of donepezil in patients with Down syndrome and Alzheimer's disease--pilot study. Int J Geriatr Psychiatry.

[44] Potter H, Woodcock JH, Boyd TD (2021). Safety and efficacy of sargramostim (GM-CSF) in the treatment of Alzheimer's disease. Alzheimers Dement (N Y).

[45] Cooper SA, Ademola T, Caslake M (2016). Towards onset prevention of cognition decline in adults with Down syndrome (The TOP-COG study): a pilot randomised controlled trial. Trials.

[46] Hanney M, Prasher V, Williams N (2012). Memantine for dementia in adults older than 40 years with Down's syndrome (MEADOWS): a randomised, double-blind, placebo-controlled trial. Lancet.

[47] Boada R, Hutaff-Lee C, Schrader A, Weitzenkamp D, Benke TA, Goldson EJ, Costa AC (2012). Antagonism of NMDA receptors as a potential treatment for Down syndrome: a pilot randomized controlled trial. Transl Psychiatry.

[48] Lott IT, Doran E, Nguyen VQ, Tournay A, Head E, Gillen DL (2011). Down syndrome and dementia: a randomized, controlled trial of antioxidant supplementation. Am J Med Genet A.

[49] Cui MY, Lin Y, Sheng JY, Zhang X, Cui RJ (2018). Exercise intervention associated with cognitive improvement in Alzheimer's disease. Neural Plast.

[50] Ptomey LT, Szabo AN, Willis EA, Gorczyca AM, Greene JL, Danon JC, Donnelly JE (2018). Changes in cognitive function after a 12-week exercise intervention in adults with Down syndrome. Disabil Health J.

[51] Castellani RJ, Plascencia-Villa G, Perry G (2019). The amyloid cascade and Alzheimer's disease therapeutics: theory versus observation. Lab Invest.

